# METTL3 promotes hyperoxia-induced pyroptosis in neonatal bronchopulmonary dysplasia by inhibiting ATG8-mediated autophagy

**DOI:** 10.1016/j.clinsp.2023.100253

**Published:** 2023-07-19

**Authors:** Lili Xu, Zhan Shi, Zhaojun Pan, Rong Wu

**Affiliations:** Huai'an Maternity and Child Healthcare Hospital, Yangzhou University Medical College, Neonatal Medical Center, Huai'an, China

**Keywords:** Bronchopulmonary Dysplasia, Neonatal, Genetics, N6-methyladenosine

## Abstract

**Objectives:**

N6-Methyladenosine (m6A) modification plays a vital role in lung disorders. However, the potential of m6A in neonatal Bronchopulmonary Dysplasia (BPD) has not been reported. This study aimed to investigate the roles of METTL3 in BPD.

**Methods:**

BPD models were established by hyperoxia in vivo and in vitro. Histological analysis was determined using HE staining. Gene expression was determined using Western blotting, qRT-PCR, and immunofluorescence. The release of IL-1β and IL-18 was detected using ELISA. The m6A sites of ATG8 were predicted by SCRAPM and verified by MeRIP assay. The location of GSDMD and ATG8 was determined by FISH assay. The interaction between ATG8 and GSDMD was detected using Coimmunoprecipitation (Co-IP). Cell pyroptosis was determined using flow cytometry and TUNEL assays.

**Results:**

METTL3 was overexpressed in BPD, which was accompanied by an increase in m6A levels. Interestingly, METTL3 suppressed hyperoxia-mediated damage and pyroptosis in BEAS-2B cells and promoted cell autophagy. METTL3-mediated m6A modification of ATG8 suppressed its expression and disrupted the interaction between ATG8 and GSDMD. However, autophagy inhibition induced pyroptosis in BEAS-2B cells. In vivo assays showed that METTL3-mediated autophagy inhibition induced a decrease in the radial alveolar count and an increase in the mean linear intercept and promoted cell pyroptosis.

**Conclusion:**

In conclusion, METTL3-mediated cell pyroptosis promotes BPD by regulating the m6A modification of ATG8. This may provide new insight into the development of BPD.

## Highlights

• m6A modification participates in the development of BPD.
• METTL3 promotes the pyroptosis of BEAS-2B cells.
• ETTL3-mediated m6A modification of ATG8 disrupts the interaction between ATG8 and GSDMD.

## Introduction

Bronchopulmonary Dysplasia (BPD) is a common complication of prematurity and is characterized by damage to the alveolar structure. [1] Although great breakthroughs have been made in the past three decades, the incidence of BPD is increasing. [2] Effective therapeutic strategies for BPD are still limited, which may be due to complications in the pathogenesis of BPD. Previous studies have reported that BPD is caused by various factors, such as fetal growth restriction, oxygen toxicity, infection, inflammation, and genetic susceptibility. 3, 4, 5 Therefore, unveiling the potential underlying molecular mechanisms may reveal the Achilles’ heel of BPD.

N6-Methyladenosine (m6A) modification is deemed the most common mRNA modification in eukaryotic cells. [6] m6A modification is frequently regulated by writers (METTL3, METTL14, RBM15, WTAP, KIAA1429, etc.), “eraser” (ALKBH5, FTO, etc.), and “reader” (IGF2BPs, YTHDs, etc.). 7, 8, 9 The m6A modification modulates RNA translation, export, decay, splicing, and stabilization. [10,11] METTL3, as an m6A “writer”, plays a vital role in maintaining mRNA stability and binds to m6A-modified RNAs. [12] This m6A methylation frequently occurs around stop codons and 3’ Untranslated Regions (3’-UTRs), which participate in biological functions and pathological processes, especially lung diseases. For instance, high expression of METTL3 predicts poor survival rates in lung cancer patients. [13] METTL3-induced m6A modification of GPX4 contributes to ferroptosis in NET-induced sepsis-associated acute lung injury. [14] METTL3-mediated m6A modification of STAT2 promotes Neonatal Pneumonia (NP). [15] However, the roles of METTL3 in BPD have not been elucidated.

A pyroptosis is a form of programmed cell death characterized by the activation of inflammasomes, such as NLRPs and NLRCs. [16,17] The activation of inflammasomes recruits ASC to cleave caspase1 (canonical) or caspase4/5/11 (noncanonical), which promotes pore formation and the pro-matures of IL-1β and IL-18. [18] GSDMD, as the executioner of pyroptosis, is cleaved by caspase and granzymes, [19] which induces oligomerization of N-terminal GSDMD and formation of pores in the cell membrane. In a study by Dapaah-Siakwan et al., Ac-YVAD-CMK-mediated inhibition of caspase1/GSDMD signaling dampened the development of BPD in vivo and in vitro. [20] In this study, the authors found that METTL3 promoted hyperoxia-induced pyroptosis in BPD by inhibiting the LC3-conjugation pathway. This study aimed to investigate the potential roles of METTL3 in BPD and the underlying molecular mechanisms.

## Materials and methods

### Specimen

Blood samples were collected from 30 premature infants with BPD and 30 non-BPD age-matched controls hospitalized in Huai'an Maternity and Child Healthcare Hospital. All the procedures were performed in accordance with STARD guidelines. This study was approved by the Ethical Committee of Huai'an Maternity and Child Healthcare Hospital. This study was approved by the parents of the enrolled patients.

### Cell culture and transfection

BEAS-2B cells were purchased from ATCC. Cells were cultured in an incubator containing 12% FBS and 1% penicillin/streptomycin (Gibco) at 37 °C in CO_2_. Cells were treated with 2 mM 3-methyladenine (3-MA) for 24 h. Cells were then divided into normoxia and hyperoxia (95% O_2_) for 24 h. METTL3 #1‒2 and its Negative Control (NC), METTL3 Overexpression plasmids (OE) and empty vector, ATG8 OE and empty vector, and GSDMD OE and empty vector were provided by GeneChem, China. Cells were transfected with Lipofectamine 3000 (Invitrogen). Five microliters of Lipofectamine and 20 pM shRNA and/or ATG8/GSDMD OE were mixed for 6 h at 37 °C. Then, transfection was performed for 48 h.

### ELISA assay

The levels of IL-1β and IL-18 were measured by enzyme-linked immunosorbent assay (ELISA).

### LDH assay

The levels of LDH were measured by an LDH kit (Abcam, USA).

### qRT-PCR

RNA was extracted from cells. QuantiTect Rev. Transcription Kit (QIAGEN) was used for cDNA synthesis. PCR was conducted using a QuantiNova SYBR Green PCR Kit (QIAGEN). The RNA levels were normalized to GAPDH and calculated using the 2^−ΔΔCq^ method. The primers used in this study were as follows: METTL3 F: 5’-TTGTCTCCAACCTTCCGTAGT-3’ and R: 5’-CCAGATCAGAGAGGTGGTGTAG-3’.

### Western blot

After transfection, cells were collected and lysed. Then, total protein was collected and concentrated using a BCA kit. Protein (30 µg) was isolated using 10% SDS-PAGE at 120v. The protein was transferred onto PVDF membranes. After blocking with 5% skimmed milk, the membranes were incubated with primary antibodies such as antiLC3I/II (ab62721, 1:2000, Abcam, USA), antiATG8 (ab98830, 1:2000, Abcam, USA), antiNLRP3 (ab263899, 1:1000, Abcam, USA), antiASC (ab283684, 1:1000, Abcam, USA), anti-Caspase1 (ab179515, 1:1000, Abcam, USA), GSDMD (ab215203, 1:1000, Abcam, USA), antiβ-actin (ab8227, 1:5000, Abcam, USA) and goat-anti-rabbit antibody (ab6721, 1:5000, Abcam, USA). Finally, the bands were captured by ECL reagents and analyzed using ImageJ (V.2.3.0).

### FISH assay

Cells were seeded into a 24-well plate. After mounting on 4% paraformaldehyde, the cells were hybridized with 2 µM Cy3-labeled GSDMD and FITC-labeled ATG8 RNA probes. Then, the cells were counterstained with DAPI. Finally, images were captured using confocal microscopy (Leica, Germany).

### Immunofluorescence assay

Cells were collected, mounted on 4% paraformaldehyde and permeabilized with 0.3% Triton X-100. Afterward, cells were incubated with primary antibodies against LC3 puncta (1:2000, ab128025, Abcam USA) and Alexa Fluor fragment of goat anti-rabbit IgG (1:2000, ab150113, Abcam USA). The cells were counterstained with DAPI. Subsequently, the cells were visualized using a fluorescence microscope (Nikon, Japan).

### MeRIP

Total RNA was collected and purified to remove ribosomal RNA and contaminated DNA. Afterward, RNA was sheared into fragments and denatured. Then, RNA was incubated with protein A/G magnetic beads conjugated with anti-m6A antibody. After elution, RNA was analyzed by qRT-PCR.

### Co‐immunoprecipitation (Co‐IP)

After transfection, cell lysates were collected using a lysis buffer. Then, the lysates were incubated with anti-GSDMD, anti-ATG8 and anti-IgG primary antibodies at room temperature for 2h and secondary antibodies at 4°C overnight. Then, the complexes were mixed with anti-IgG magnetic beads, rinsed in IP buffer, and analyzed using a western blot assay.

### TUNEL assay

Cells were harvested, mounted on 4% paraformaldehyde, and permeabilized in 0.25% Triton-X100. Afterward, the cells were stained using an in-situ Cell Death Detection Kit. The images were visualized by fluorescence microscopy (Nikon, Japan). Cell death rates = TUNEL-positive cells/total cells ×100%.

### Animal models

Neonatal C57BL/6J, B6.129S4-Ccr2tm1Ifc/J (METTL3^−/−^), and the respective Wild-Type (WT) control mice were purchased from the Experimental Animal Center of Nanjing Medical University. Mice were divided into three groups: 1) C57BL/6J mice were exposed to 21% oxygen (normoxia, NRMX) for 14 days; 2) C57BL/6J, METTL3^−/−^ and WT control mice were exposed to 80% O_2_ oxygen (hyperoxia, HYRX) for 28 days; 3) Mice were injected with 15 µg of 3-MA 30 min before hyperoxia exposure. The procedures were performed in accordance with ARRIVE guidelines.

### Tissue preparation

The mice were anesthetized, and their right and left lungs were immediately stored in liquid nitrogen at -80°C. Then, the tissues were used for Histological analysis (HE), gene expression determination (ELISA and immunofluorescence), and m6A level determination (MeRIP).

### Bioinformatics analysis

The m6A modification sites and RNA secondary structure were predicted by the online database SCRAMP (http://www.cuilab.cn/sramp/).

### Statistical analysis

Data were analyzed using SPSS26.0 and presented as the mean ± SD. The differences were analyzed by Student's *t*-test and ANOVA; p < 0.05 was deemed statistically significant.

## Results

### METTL3 is overexpressed in BPD

**Fig. 1:**
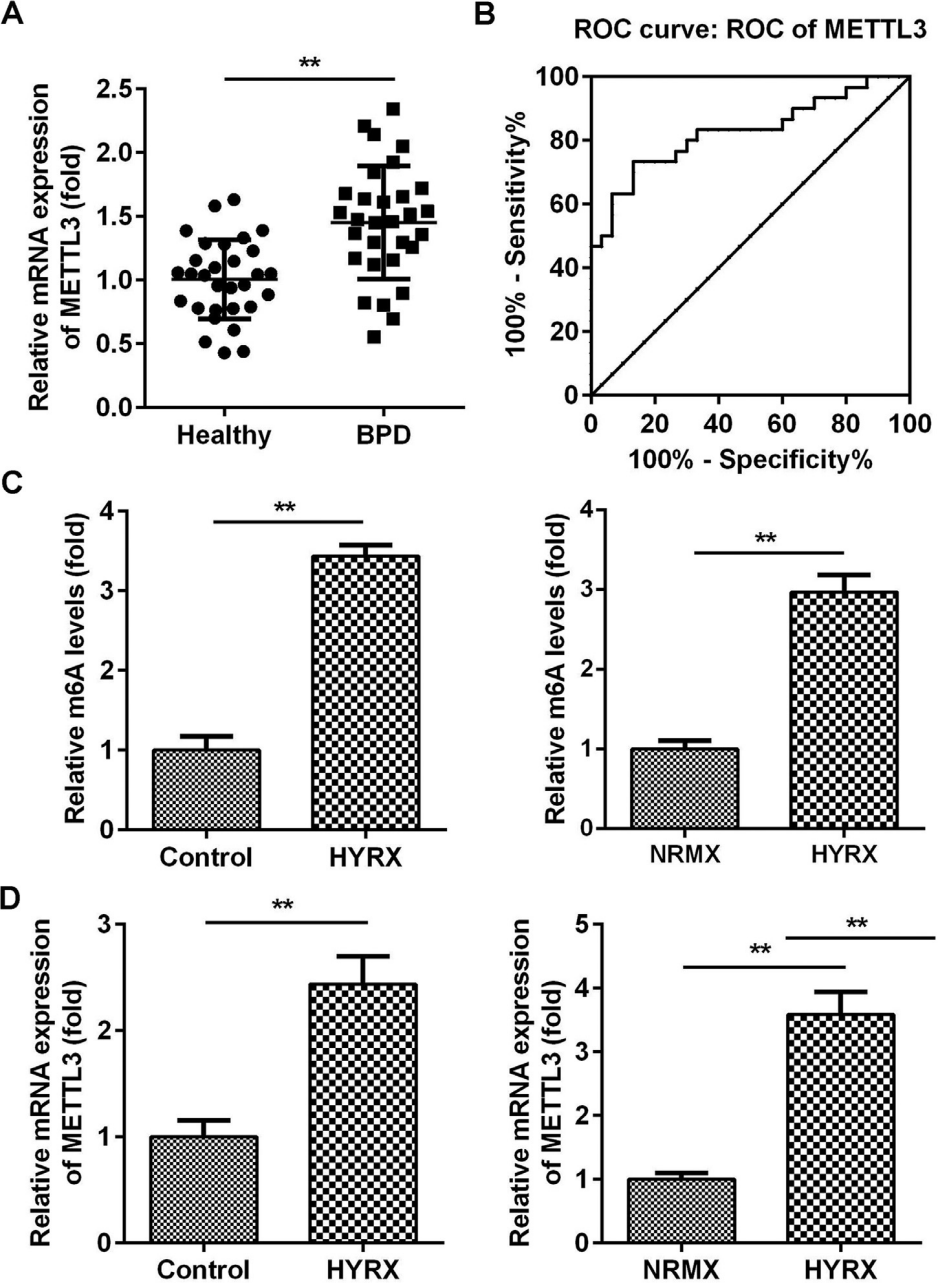
METTL3 was overexpressed in BPD. (A) The mRNA expression of METTL3 in BPD patients detected by qRT-PCR. (B) The ROC curve analysis of METTL3 expression. (C) The m6A levels in vivo and in vitro detected by MeRIP assay. (D) The mRNA expression of METTL3 in vivo and in vitro detected by qRT-PCR. **p < 0.01. Data represent at least three independent sets of experiments.

METTL3 plays a vital role in neonatal lung diseases, including BPD. Therefore, the authors determined the mRNA levels of METTL3 in BPD patients. The authors found that METTL3 was markedly increased in BPD patients compared with healthy controls (Fig. 1A). The Receiver Operating Characteristic curve (ROC) showed that the area under the ROC curve was 0.8267, and the 95% Confidence Interval was between 0.7193 and 0.9340, suggesting that METTL3 can be a sensitive biomarker for BPD (Fig. 1B). To further verify this, the authors determined the expression of METTL3 *in vivo* and *in vitro*. As shown in Fig. 1C, METTL3 mRNA expression was significantly increased under hyperoxic conditions. METTL3 is an m6A reader, the overexpression of which is frequently accompanied by an increase in total m6A levels. MeRIP assays showed that the m6A levels in vivo and in vitro were also increased under hyperoxic conditions (Fig. 1D). These findings suggest that a high level of METTL3 may contribute to the pathogenesis of BPD.

### METTL3 knockdown suppresses the pyroptosis of BEAS-2B cells

**Fig. 2:**
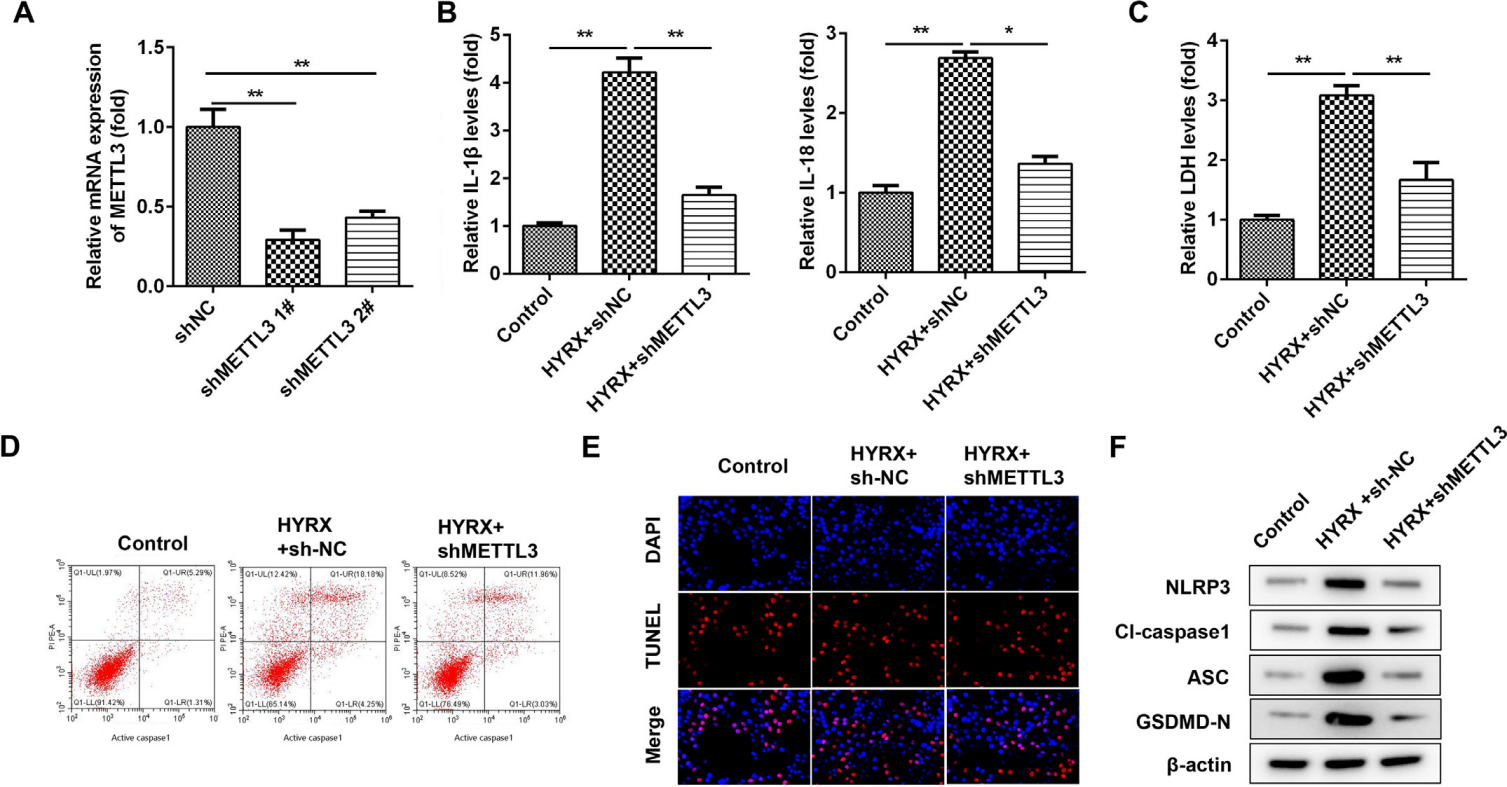
METTL3 knockdown suppresses the pyroptosis of BEAS-2B cells. (A) METTL3 mRNA expression detected by western blot. (B) The release of IL-1β and IL-18 detected by ELISA. (C) The release of LDH. (D) Cell pyroptosis determined by flow cytometry. (E) Cell pyroptosis determined by TUNEL assay. (F) Pyroptosis-related protein expression detected by western blot. **p < 0.01. Data represent at least three independent sets of experiments.

To further verify the roles of METTL3, the authors determined the cellular functions of BEAS-2B cells after transfection with shMETTL3. Fig. 2A shows that shMETTL3 decreased METTL3 mRNA expression, suggesting successful transfection. shMETTL3 #1, with more potent efficiency, was applied for the following experiment. METTL3 knockdown notably decreased the release of IL-1β and IL-18 (Fig. 2B). Moreover, METTL3 knockdown decreased the release of LDH induced by hyperoxia (Fig. 2C). Additionally, hyperoxia-induced cell death was significantly alleviated by METTL3 knockdown (Fig. 2 D and E). Meanwhile, METTL3 deficiency suppressed the expression of NLRP3, cleaved caspase-1 and GSDMD (Fig. 2F).

### METTL3 knockout promotes autophagy in BEAS-2B cells

**Fig. 3:**
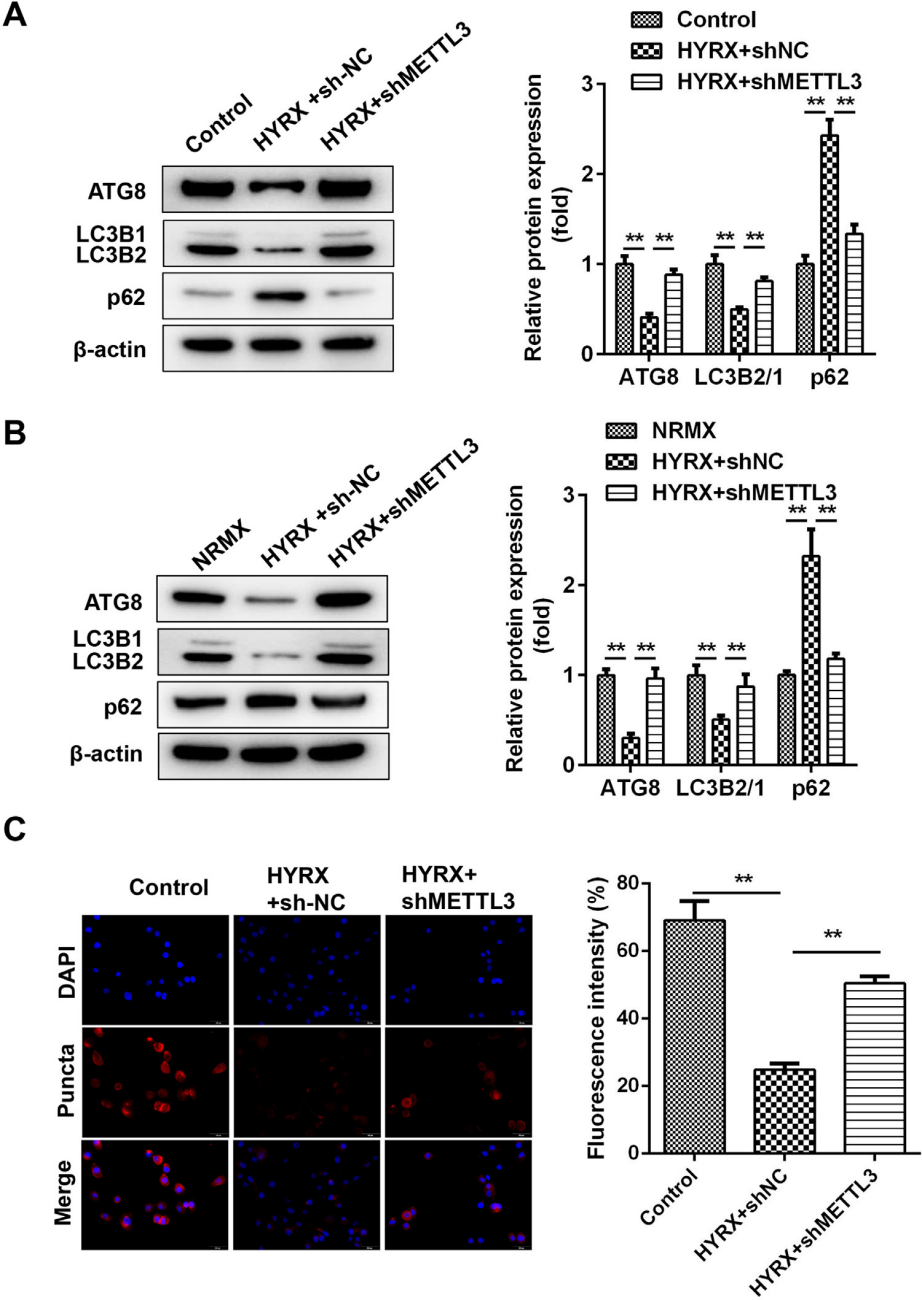
METTL3 knockout promotes the autophagy of BEAS-2B cells. (A) Autophagy-related protein expression in vivo detected by western blot. (B) Autophagy-related protein expression in vitro detected by western blot. (C) Autophagy-related protein expression in vitro detected by immunofluorescence **p < 0.01. Data represent at least three independent sets of experiments.

Previous studies have shown that METTL3 regulates pyroptosis by regulating autophagy. [21] Whether METTL3 regulates the pyroptosis of BEAS-2B cells by inhibiting autophagy. In vivo assays showed that hyperoxia decreased the protein expression of ATG8 and LC3II/I but increased p62, which was abrogated by METTL3 deficiency (Fig. 3A). This is consistent with the results from the in vitro assay (Fig. 3B). Moreover, immunofluorescence showed that LC3 puncta were markedly increased in the shMETTL3 group (Fig. 3C).

### METTL3 mediates the m6A modification of ATG8

**Fig. 4:**
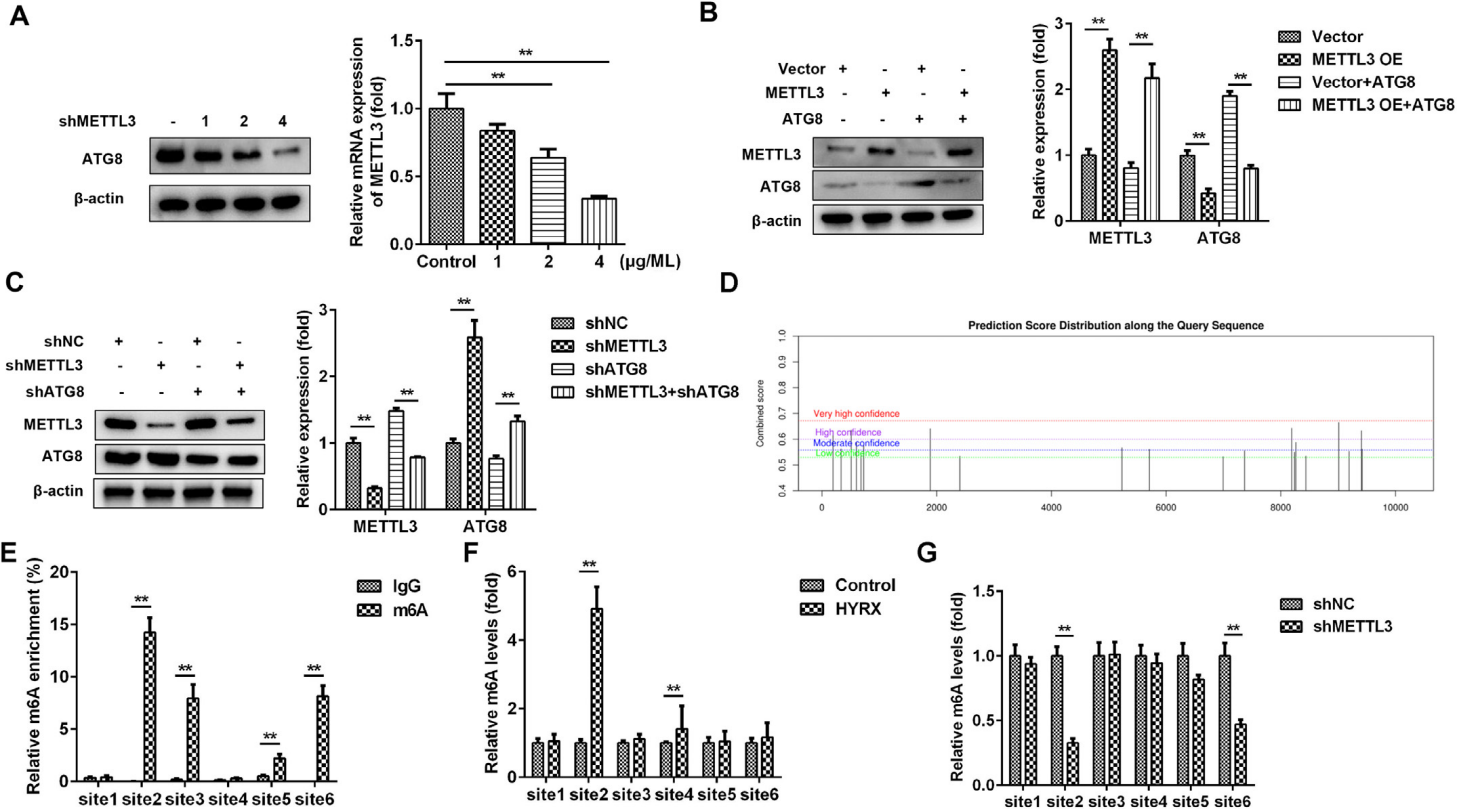
METTL3 mediates the m6A modification of ATG8. (A‒C) ATG8 protein expression detected by western blot. (D) The potential m6A modification sites were predicted by SCRAMP. (E‒G) The potential m6A modification sites verified by MeRIP assay. **p < 0.01. Data represent at least three independent sets of experiments.

METTL3, as an m6A reader, induces RNA decay via m6A modification of its target genes. Then, the authors postulated that METTL3 may inhibit autophagy by mediating m6A modification of its target in LC3 signaling. Therefore, METTL3 may mainly regulate ATG8 in this study. To further verify this, cells were transfected with 1, 2 and 4 µg/mL METTL3. The results showed that ATG8 protein expression was decreased by METTL3 in a dose-dependent manner (Fig. 4A). Moreover, the METTL3-mediated decrease in ATG8 protein expression was alleviated by ATG8 overexpression (Fig. 4B), while METTL3 knockdown-induced upregulation of ATG8 was abated by shATG8 (Fig. 4C), suggesting that METTL3 negatively regulated the expression of ATG8. Additionally, the authors analyzed the possible m6A sites of ATG8 and found that there were 6 high confidence sites (Fig. 4D). The MeRIP assay showed that m6A was enriched at sites 2, 3, 5, and 6 (Fig. 4E). Moreover, the m6A levels of ATG8 were significantly increased at site 2 and site 4 under hyperoxia conditions (Fig. 4F) but markedly decreased at site 2 and site 6 after transfection with shMETTL3 (Fig. 4G). Therefore, METTL3 may regulate the m6A modification of ATG8 at site 2.

### METTL3 disrupts the interaction between GSDMD and ATG8

**Fig. 5:**
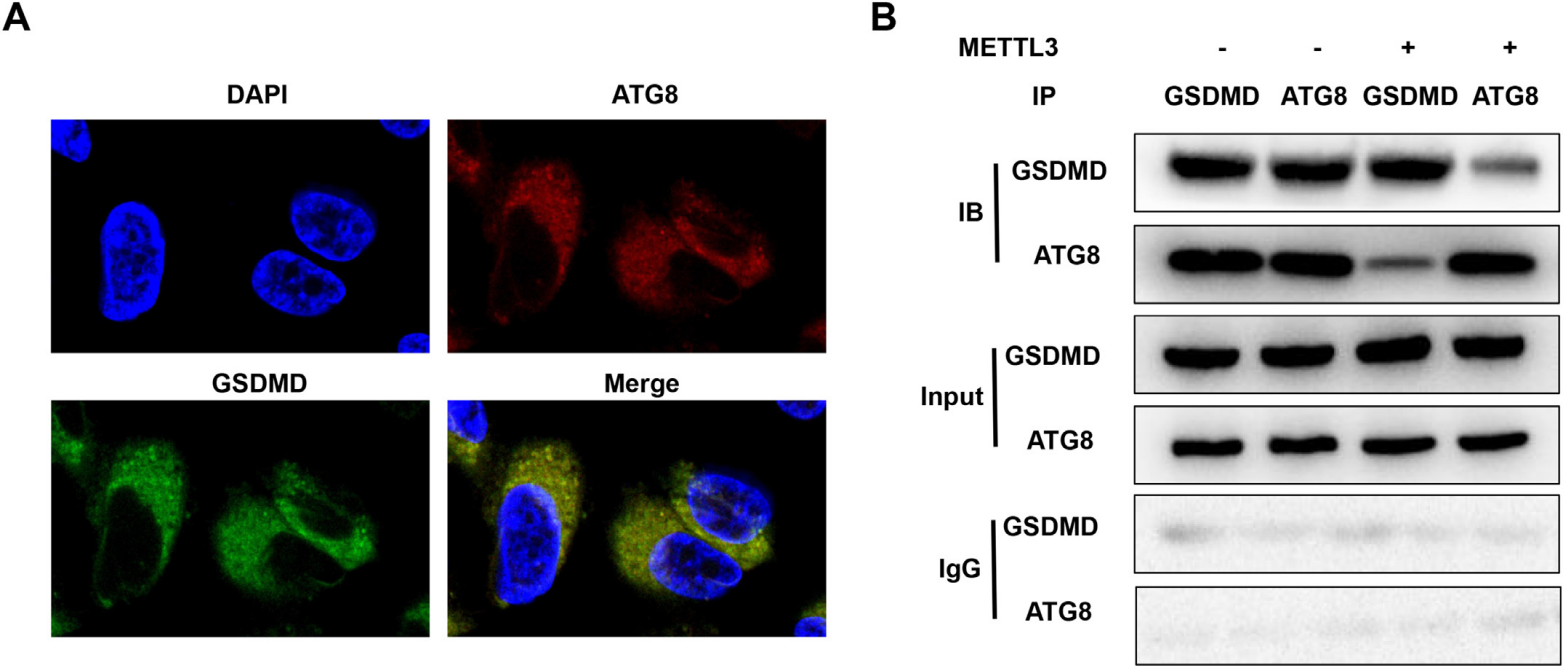
METTL3 disrupts the interaction between GSDMD and ATG8. (A) The co-localization of GSDMD and ATG8 was detected by FISH assay. (B) The interaction between GSDMD and ATG8 detected by Co-IP. Data represent at least three independent sets of experiments.

ATGs are required for inflammation resolution and play a vital role in integral membrane proteins. [22] Therefore, METTL3-mediated m6A modification of ATG8 may induce the decay of ATG8, disrupt the interaction between ATG8 and the membrane protein GSDMD, and suppress its capacity to resolve inflammation. FISH assays showed that ATG8 was colocalized in the cytoplasm of BEAS-2B cells (Fig. 5A). However, METTL3 overexpression blocked the interaction between ATG8 and GSDMD (Fig. 5B).

### Autophagy inhibition promotes pyroptosis in BEAS-2B cells

**Fig. 6:**
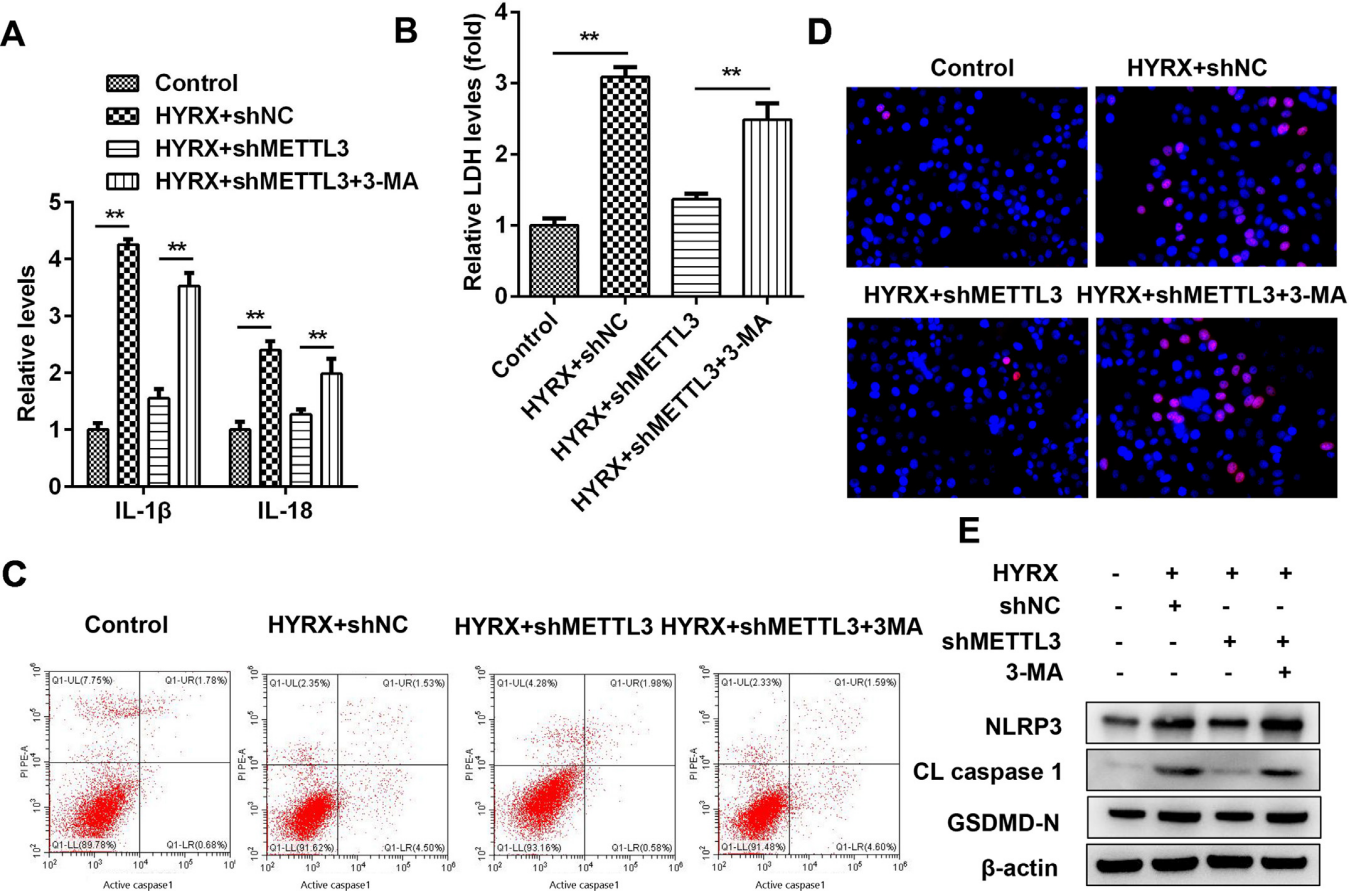
Autophagy inhibition promotes the pyroptosis of BEAS-2B cells. (A) The release of IL-1β and IL-18 detected by ELISA. (B) The release of LDH. (C) Cell pyroptosis determined by flow cytometry. (D) Cell pyroptosis determined by TUNEL assay. (E) Pyroptosis-related protein expression detected by western blot. **p < 0.01. Data represent at least three independent sets of experiments.

Figs. 3 and 5 show that autophagy may participate in METTL3-mediated pyroptosis. To further verify this, cells were exposed to 3-MA. The authors found that autophagy inhibition increased the release of proinflammatory cytokines (IL-1β and IL-18) (Fig. 6A) and promoted cytotoxicity (Fig. 6B). Moreover, 3-MA exposure promoted the pyroptosis of BEAS-2B cells (Fig. 6 C‒E).

### METTL3 knockout protects against pyroptosis in vivo via autophagy inhibition

**Fig. 7:**
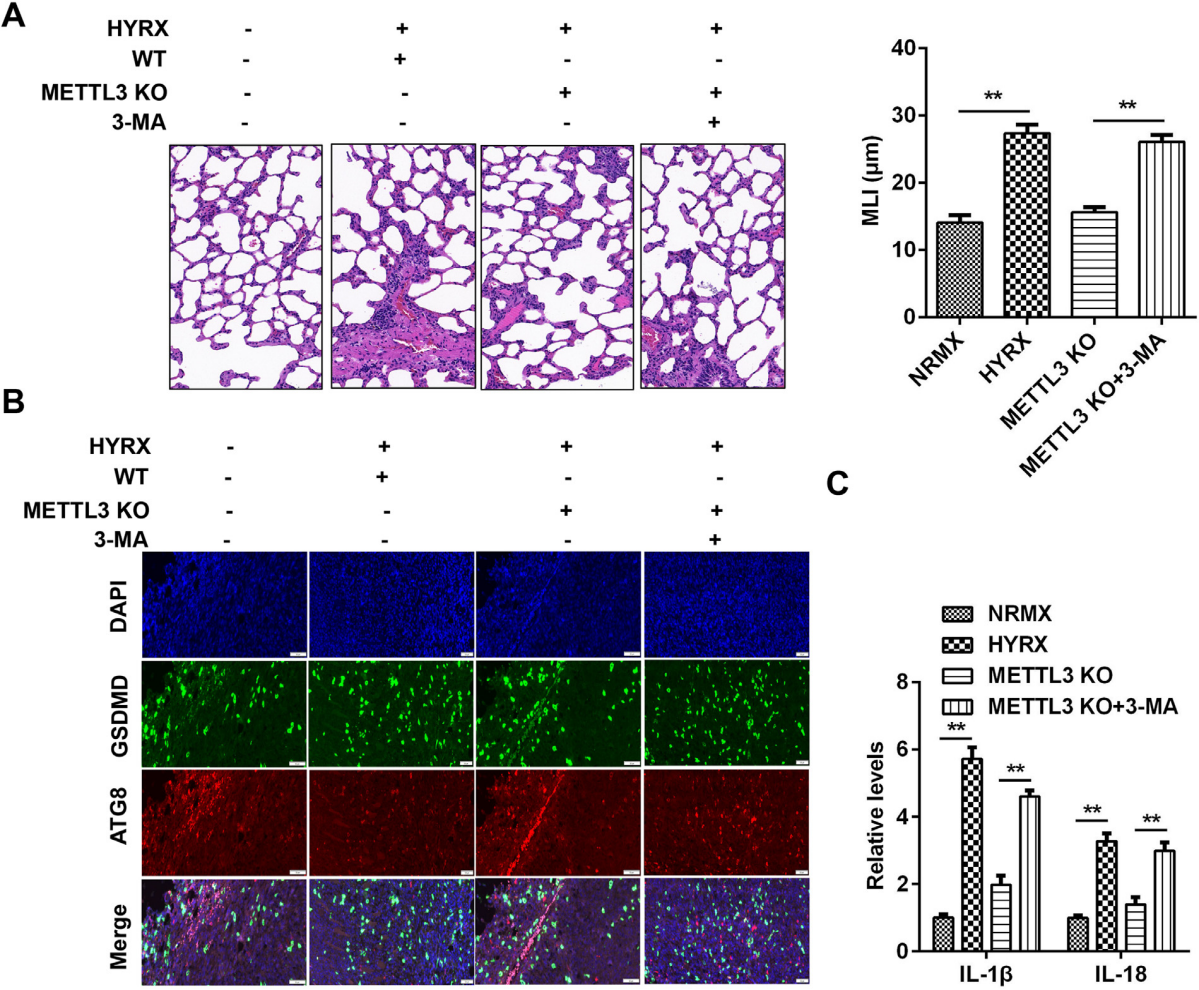
METTL3 knockout suppresses protected against pyroptosis in vivo. (A) Histological analysis performed using HE staining. (B) ATG8 and GSDMD expression detected by immunofluorescence. (C) The release of IL-1β and IL-18 detected by ELISA. **p < 0.01. Data represent at least three independent sets of experiments.

To further verify the roles of METTL3 in BPD, the authors performed an *in vivo* assay. As shown in Fig. 7A, hyperoxia exposure induced severe impairment of alveolar growth, large airspaces, and incomplete alveolar septation; however, METTL3 knockout improved alveolarization and restored lung architecture, which was abrogated by 3-MA. Moreover, METTL3 knockout increased the expression of ATG8 and decreased GSDMD, which was dampened by autophagy inhibition (Fig. 7B). Additionally, the METTL3 knockout-mediated suppression of the inflammatory response was abated by 3-MA (Fig. 7C), suggesting that METTL3 knockout protected against inflammation-induced pyroptosis *in vivo*.

## Discussion

In this study, METTL3 was overexpressed in BPD. METTL3 knockdown suppressed hyperoxia-induced pyroptosis in BPD in vivo and in vitro, while autophagy inhibition promoted pyroptosis. Moreover, METTL3 mediated the modification of ATG8 and blocked the interaction between ATG8 and GSDMD. To our knowledge, this is the first study to investigate the potential of m6A modification and METTL3 in BPD. The findings in this study may lay the foundation for further study.

Hyperoxia-induced oxygen toxicity contributes to uninhibited inflammation and interrupted lung development. [23] The hyperoxia-stimulated inflammatory cascade results in the influx of proinflammatory cells (such as neutrophils and macrophages) as well as the release of proinflammatory cytokines. [24] In this study, hyperoxia exposure induced cytotoxicity and the release of IL-1β and IL-18. Continuous inflammation can induce programmed cell death: pyroptosis and necroptosis. However, pyroptosis is differentiated from necroptosis: (1) Necroptosis is frequently found as a backup cell death defense mechanism that is triggered when apoptosis is hindered, while pyroptosis is a primary cellular response following the sensing of potentially damaging insults (including hyperoxia, DAMPs); [25] (2) Necroptosis is characterized by the activation of RIPK1/RIPK3 signaling and the assembly of necrosomes, while pyroptosis is characterized by GSDM cleavage and inflammasome formation (such as NLRP3). [26] Therefore, in this study, hyperoxia-induced cell death occurred via pyroptosis.

METTL3 is abnormally expressed in lung diseases. For instance, METTL3-mediated m6A modification promotes idiopathic pulmonary fibrosis. [27] METTL3 interacts with YTHDF1 to induce the translation of MAGED1 and the pathogenesis of pulmonary hypertension. [28] METTL3 promotes inflammation and the development of neonatal pneumonia. [15] These findings suggest that METTL3 is frequently overexpressed in lung diseases and that high levels of METTL3 are associated with the initiation and development of lung diseases. However, the role of METTL3 in lung cancer is elusive. METTL3 functions as an oncogene in non-small cell lung cancer as well as a tumor suppressor in lung adenocarcinoma, [29,30] suggesting that the role of METTL3 varies with the subtypes of lung cancer. Therefore, identifying the role of METTL3 in BPD is of vital importance. In this study, METTL3 was overexpressed in BPD in vivo and in vitro. Interestingly, METTL3 deficiency suppressed pyroptosis and promoted cell autophagy. Nevertheless, METTL3 is involved in cellular functions by regulating its targets. METTL3 modulates RNA translation, decay, splicing and stability. [10,11] For instance, METTL3 mediates m6A modification of circIGF2BP3 and promotes its circularization and stability. [31] METTL3 mediates m6A modification of TFEB and decreases its expression. [32] In this study, METTL3 suppressed ATG8-mediated autophagy by inhibiting its expression, which is consistent with the findings of Chen et al. [33] These findings further verify that METTL3 may play a passive role in BPD.

Autophagy, with “mission” defense, metabolism, and quality control, plays a fundamental role in maintaining intracellular homeostasis. [34] A failure in autophagy functions is frequently manifested as the accumulation of inflammation. [35] In this study, METTL3-induced inhibition of autophagy was accompanied by the release of proinflammatory cytokines, suggesting the loss of self-repair capacity. ATG8, as a member of the ATG family, is indispensable in the execution of autophagy, including phagophore initiation, expansion, transition, and fusion with lysosomes. [36] Moreover, ATG8, with the capability to regulate membrane fusion events, is downregulated in LPS-induced noncanonical pyroptosis, [37,38] suggesting that ATG8 deficiency-mediated autophagy inhibition may be closely related to inflammation-induced cell death or pyroptosis. In this study, METTL3-mediated m6A modification of ATG8 suppressed its expression and disrupted the interaction between ATG8 and GSDMD, which contributed to pore formation in membranes and the release of IL-1β and IL-18. Therefore, METTL3 promoted hyperoxia-induced pyroptosis in BPD by suppressing ATG8-mediated autophagy.

In conclusion, METTL3-mediated m6A modification of ATG8 decreased its expression and blocked the ATG8-GSDMD interaction, which promoted pyroptosis in BPD. This may provide a novel strategy for BPD.

## Ethical approval

This study was approved by the Ethical Committee of Huai'an Maternity and Child Healthcare Hospital (nº F2022035).

## Data availability

The data generated in the present study may be requested from the corresponding author.

## Funding

This research was funded by grants fromJiangsu Provincial Health and Family Planning Commission(Project N.: H2018009,F201812)

## CRediT authorship contribution statement

**Lili Xu:** Formal analysis, Data curation, Writing – original draft. **Zhan Shi:** Formal analysis, Data curation, Writing – original draft. **Zhaojun Pan:** Data curation, Writing – review & editing. **Rong Wu:** Conceptualization.

## Declaration of Competing Interest

The authors declare no conflicts of interest.

## Appendix Supplementary materials

Image, application 1
