# Supplementary material for: METTL3 promotes hyperoxia-induced pyroptosis in neonatal bronchopulmonary dysplasia by inhibiting ATG8-mediated autophagy
Source: Clinics (Sao Paulo). 2023 Jul 19;78:100253. doi: 10.1016/j.clinsp.2023.100253 (PMC10387564; doi:10.1016/j.clinsp.2023.100253)
Supplement: Supplementary file 1 [file mmc1.docx]

CLINICS-D-23-00073_Suplementary Material

**Supplemental Table 1** The methods used.

| **Methods** | **Procedures** |
| --- | --- |
| Specimen | Blood samples were collected from 30 premature infants with BPD and 30 non-BPD age-matched controls hospitalized in Huai’an Maternity and Child Healthcare Hospital. This study was approved by the Ethical Committee of Huai’an Maternity and Child Healthcare Hospital. This study was approved by the parents of the enrolled patients. |
| Cell culture and transfection | BEAS-2B cells were purchased from ATCC. Cells were cultured in an incubator containing 12% FBS and 1% penicillin/streptomycin (Gibco) at 37°C in CO_2_. Cells were treated with 2 mM 3-methyladenine (3-MA) for 24h. Cells were then divided into normoxia and hyperoxia (95% O_2_) for 24h. METTL3 #1‒2 and its negative control (NC), METTL3 Overexpression plasmids (OE) and empty vector, ATG8 OE and empty vector, and GSDMD OE and empty vector were provided by GeneChem, China. Cells were transfected with Lipofectamine 3000 (Invitrogen). Five microliters of Lipofectamine and 20 pM shRNA and/or ATG8/GSDMD OE were mixed for 6h at37°C. Then, transfection was performed for 48h. |
| ELISA assay | The levels of IL-1β and IL-18 were measured by Enzyme-Linked Immunosorbent Assay (ELISA). |
| LDH assay | The levels of LDH were measured by an LDH kit (Abcam, USA). |
| qRT‒PCR | RNA was extracted from cells. QuantiTect Rev. Transcription Kit (QIAGEN) was used for cDNA synthesis. PCR was conducted using a QuantiNova SYBR Green PCR Kit (QIAGEN). The RNA levels were normalized to GAPDH and calculated using the 2^-ΔΔCq^ method. The primers used in this study were as follows: METTL3 F: 5’-TTGTCTCCAACCTTCCGTAGT-3’ and R: 5’-CCAGATCAGAGAGGTGGTGTAG-3’. |
| Western blot | After transfection, cells were collected and lysed. Then, total protein was collected and concentrated using a BCA kit. Protein (30 µg) was isolated using 10% SDS-PAGE at 120v. The protein was transferred onto PVDF membranes. After blocking with 5% skimmed milk, the membranes were incubated with primary antibodies such as antiLC3I/II (ab62721, 1: 2000, Abcam, USA), antiATG8 (ab98830, 1:2000, Abcam, USA), antiNLRP3 (ab263899, 1:1000, Abcam, USA), antiASC (ab283684, 1:1000, Abcam, USA), anti-Caspase1 (ab179515, 1:1000, Abcam, USA), GSDMD (ab215203, 1:1000, Abcam, USA), antiβ-actin (ab8227, 1:5000, Abcam, USA) and goat-anti-rabbit antibody (ab6721, 1:5000, Abcam, USA). Finally, the bands were captured by ECL reagents and analyzed using ImageJ (V.2.3.0). |
| FISH assay | Cells were seeded into a 24-well plate. After mounting on 4% paraformaldehyde, the cells were hybridized with 2 µM Cy3-labeled GSDMD and FITC-labeled ATG8 RNA probes. Then, the cells were counterstained with DAPI. Finally, images were captured using confocal microscopy (Leica, Germany). |
| Immunofluorescence assay | Cells were collected, mounted on 4% paraformaldehyde and permeabilized with 0.3% Triton X-100. Afterward, cells were incubated with primary antibodies against LC3 puncta (1: 2000, ab128025, Abcam USA) and Alexa Fluor fragment of goat anti-rabbit IgG (1:2000, ab150113, Abcam USA). The cells were counterstained with DAPI. Subsequently, the cells were visualized using a fluorescence microscope (Nikon, Japan). |
| MeRIP | Total RNA was collected and purified to remove ribosomal RNA and contaminated DNA. Afterward, RNA was sheared into fragments and denatured. Then, RNA was incubated with protein A/G magnetic beads conjugated with anti-m6A antibody. After elution, RNA was analyzed by qRT-PCR. |
| Co‐immunoprecipitation (Co‐IP) | After transfection, cell lysates were collected using lysis buffer. Then, the lysates were incubated with anti-GSDMD, anti-ATG8 and anti-IgG primary antibodies at room temperature for 2h and secondary antibodies at 4°C overnight. Then, the complexes were mixed with anti-IgG magnetic beads, rinsed in IP buffer, and analyzed using a western blot assay. |
| TUNEL assay | Cells were harvested, mounted on 4% paraformaldehyde, and permeabilized in 0.25% Triton-X100. Afterward, the cells were stained using an in-situ Cell Death Detection Kit. The images were visualized by fluorescence microscopy (Nikon, Japan). Cell death rates = TUNEL-positive cells/total cells ×100%. |
| Animal models | Neonatal C57BL/6J, B6.129S4-Ccr2tm1Ifc/J (METTL3^−/−^) and the respective Wild-Type (WT) control mice were purchased from the Experimental Animal Center of Nanjing Medical University. Mice were divided into three groups: 1) C57BL/6J mice were exposed to 21% oxygen (normoxia, NRMX) for 14 days; 2) C57BL/6J, METTL3^−/−^ and WT control mice were exposed to 80% O_2_ oxygen (hyperoxia, HYRX) for 28 days; 3) Mice were injected with 15 µg of 3-MA 30 min before hyperoxia exposure. |
| Tissue preparation | The mice were anesthetized, and their right and left lungs were immediately stored in liquid nitrogen at -80°C. Then, the tissues were used for Histological analysis (HE), gene expression determination (ELISA and immunofluorescence), and m6A level determination (MeRIP). |
| Bioinformatics analysis | The m6A modification sites and RNA secondary structure were predicted by the online database SCRAMP (http://www.cuilab.cn/sramp/). |
| Statistical analysis | Data were analyzed using SPSS26.0 and presented as the mean ± SD. The differences were analyzed by Student’s *t*-test and ANOVA; p < 0.05 was deemed statistically significant. |
